# Supplementary material for: An Empirical Investigation of “Physician Congestion” in U.S. University Hospitals
Source: Int J Environ Res Public Health. 2019 Mar 2;16(5):761. doi: 10.3390/ijerph16050761 (PMC6427243; doi:10.3390/ijerph16050761)
Supplement: Supplementary file 1 [file ijerph-16-00761-s001.pdf]

Table S1. The details of the U.S.-based university hospitals sampled in the study.

Hospital	City	State
Cleveland Clinic	Cleveland	OH
Johns Hopkins Hospital	Baltimore	MD
Massachusetts General Hospital	Boston	MA
New York-Presbyterian University Hospital of Columbia & Cornell	NYC	NY
Brigham and Women's Hospital	Boston	MA
Ronald Reagan UCLA Medical Center Los Angeles	Los Angeles	CA
Duke University Medical Center	Durham	NC
Hospital of the University of Pennsylvania Philadelphia	Philadelphia	PA
University of Michigan Hospitals and Health Centers	Ann Arbor	MI
Northwestern Memorial Hospital	Chicago	IL
UPMC-University of Pittsburgh Medical Center	Pittsburgh	PA
Vanderbilt University Medical Center	Nashville	TN
Loyola University Medical Center	Maywood	IL
University of Alabama Hospital at Birmingham	Birmingham	AL
Hahnemann University Hospital	Philadelphia	PA
University of Kansas Hospital	Kansas City	KS
Harper University Hospital Detroit	Detroit	MI
Hackensack University Medical Center	Hackensack	NJ
Wake Forest University Baptist Medical Center	Winston-Salem	NC
University of California San Francisco Medical Center	San Francisco	CA
Stony Brook University Hospital	Stony Brook	NY
Robert Wood Johnson University Hospital	New Brunswick	NJ
University of Minnesota Medical Center	Minneapolis	MN
Baylor University Medical Center	Dallas	TX
University of Wisconsin Hospital and Clinics	Madison	WI
University Hospitals Case Medical Center	Cleveland	OH
Oregon Health and Science University	Portland	OR
Dartmouth-Hitchcock Medical Center	Lebanon	NH
University of California Davis Medical Center	Sacramento	CA
University of Virginia Medical Center	Charlottesville	VA
University of Rochester Medical Center	Rochester	NY
University Hospital of Brooklyn-SUNY Downstate Medical Center	Brooklyn	NY
Loma Linda University Medical Center	Loma Linda	CA
St. Louis University Hospital	Saint Louis	MO
North Shore University Hospital Manhasset	Manhasset	NY
Memorial University Medical Center	Savannah	GA
University of Kentucky Chandler Hospital	Lexington	KY
Penn State Milton S. Hershey Medical Center	Hershey	PA
Boston Medical Center	Boston	MA
Staten Island University Hospital	Staten Island	NY
George Washington University Hospital	Washington	DC
University of Utah Health Care	Salt Lake City	UT
West Virginia University Hospitals	Morgantown	WV
University Community Hospital	Tampa	FL
North shore University Hospital	Evanston	IL
OSF St. Francis Medical Center	Peoria	IL
Gunderson Lutheran La Crosse	La Crosse	WI
SUNY Upstate Medical University	Syracuse	NY
University Medical Center	Lubbock	TX
University of Missouri Health Care Columbia	Columbia	MO

Table S2. Regression results: Dependent variable: Ln (*IHQ*)[1] including interaction effects with Research impact.

**Variables**	**Model (1) PBR**	**Model (2) PBR_FTEs**
PBR	2.251**	3.724**
	(2.27)	(2.11)
PBR^2	-2.629*	-13.688**
	(-1.86)	(-1.87)
PBR*H-index	0.489**	0.127**
	(2.16)	(2.84)
PBR^2 *H-index	-1.17**	-0.846**
	(-1.96)	(-1.98)
Orthopedics	-0.02	0.004
	(-0.37)	(0.08)
Cardiology	0.051	0.064
	(0.74)	(0.91)
For-Profit	-0.040	-0.108**
	(-0.69)	(-2.00)
Length of stay	-0.014	0.051
	(-0.4)	(1.63)
Ln(Median income)	-0.024	0.081
	(-0.26)	(0.95)
Over 65	0.192	0.232
	(0.18)	(0.27)
Cons	3.110***	2.477**
	(3.11)	(2.59)

Notes. * $p < 0.10$, ** $p < 0.05$, *** $p < 0.01$, t-statistics in parentheses, (1) The variables *PBR*/ *PBR_FTEs* were calculated from first-stage results of the GMM-IV regression in Table 2.

Table S3. IV- GMM -regression. Dependent variable *Survival* rate. Endogenous variable *PBR*.

	Model 1 ***PBR***	Model 2 ***PBR_FTEs***
PBR	1.139**	PBR_FTEs 2.332**
	(1.98)	(1.94)
PBR^2	-1.446*	$(PBR\_FTEs)^2$ -4.871*
	(-1.68)	(-1.66)
Orthopedics	-0.044	-0.043
	(-0.88)	(-0.88)
Cardiology	-0.062	-0.066
	(-1.18)	(-1.26)
For-Profit	0.032	0.035
	(0.75)	(0.80)
Length of stay	-0.017	-0.014
	(-0.67)	(-0.40)
Ln(Median income)	-0.060	-0.101
	(-0.82)	(-1.34)
Over 65	-1.078	-0.947
	(-1.45)	(-1.22)
Revenue	3.68e-05	5.04e-10*
	(1.41)	(1.82)
Clinical services	0.017***	0.016***
	(3.75)	(3.42)
Research impact	0.020*	0.089**
	(1.89)	(2.02)
Research volume	-0.025	-0.015
	(-0.76)	(-0.48)
Cons	2.120***	2.454***
	(2.74)	(3.14)
Adj-R2	0.115	0.198
N	148	148
Optimal PBR	**0.41**	**0.23**

Notes: *t* statistics in parentheses; * $p < 0.10$, ** $p < 0.05$, *** $p < 0.01$; (1) Durbin and Wu-Hausman test for the endogeneity of PBR//*PBR_FTEs* rejected the null hypothesis of exogeneity (P<0.0000). The under-identification test was significant (2) Utest confirmed the existence of an inverted U-shape with respect to the *PBR* at the 5% level.

Table S4. Regressions results for three dependent variables: Hospital's total revenue, net income/loss, NIH funds [4].

	(1) Ln(net income/loss)[1]	(2) Ln(tot revenue)[1]	(3) Ln(NIH awards)[2,3]
PBR	0.003***	0.054***	0.339***
	(4.90)	(4.88)	(3.15)
PBR^2	-0.003***	-0.064***	-0.284***
	(-4.09)	(-4.02)	(-2.71)
Orthopedics	-0.002	-0.038	0.137
	(-0.47)	(-0.47)	(0.12)
Cardiology	-0.008**	-0.176**	-1.561
	(-2.06)	(-2.11)	(-1.38)
For-Profit	0.002	0.026	-0.438
	(0.32)	(0.33)	(-0.43)
Length of stay	-0.0004	-0.011	-2.697***
	(-0.19)	(-0.23)	(-4.26)
ln(Median income)	-0.007	-0.160	-5.045***
	(-1.28)	(-1.42)	(-3.64)
Over 65	0.134**	2.950**	-12.56
	(2.32)	(2.38)	(-0.77)
Clinical services	0.002***	0.047***	-0.287***
	(6.37)	(6.38)	(-2.92)
Patient days	1.21E-07***	2.65E-06***	2.45E-05***
	(6.99)	(7.15)	(7.32)
Research impact	0.0002	0.004	0.326
	(0.18)	(0.18)	(1.15)
Research volume	0.002	0.052	0.441
	(0.95)	(0.99)	(0.87)
Cons.	3.009***	20.39***	75.41***
	(53.27)	(16.79)	(4.80)
**Optimal ratio**	**0.41**	**0.42**	**0.60**
Adj-R2	0.654	0.660	0.310
N	149	149	149

Notes: *t* statistics in parentheses; * $p < 0.10$, ** $p < 0.05$, *** $p < 0.01$; [1] Exogeneity test for PBR and PBR^2 was rejected, thus Stata 14.1 ivreg2 command was employed. [2] Exogeneity test for PBR and PBR^2 was not rejected, we thus employed a simple OLS with heteroscedasticity-consistent standard errors. [3] Data source for NIH funds: https://www.report.nih.gov/award/index.cfm; [4] Since the dependent variables are not given only at the ward level, we could only test the effect of PBR at the hospital level).

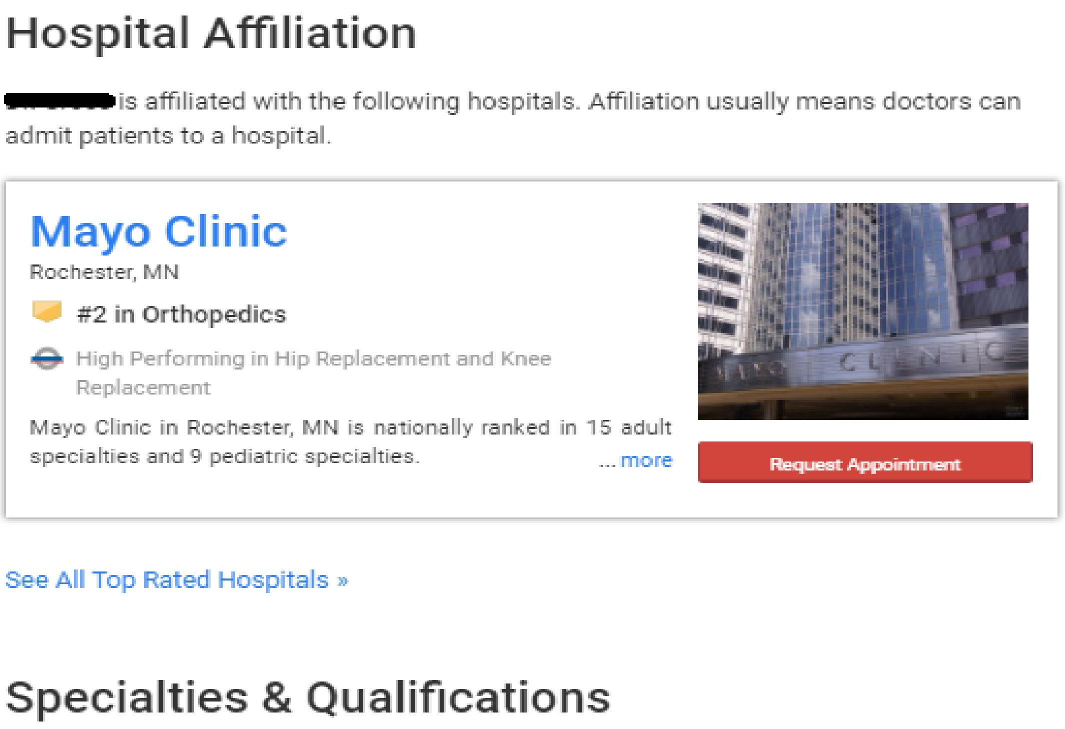

Physician with one affiliation.

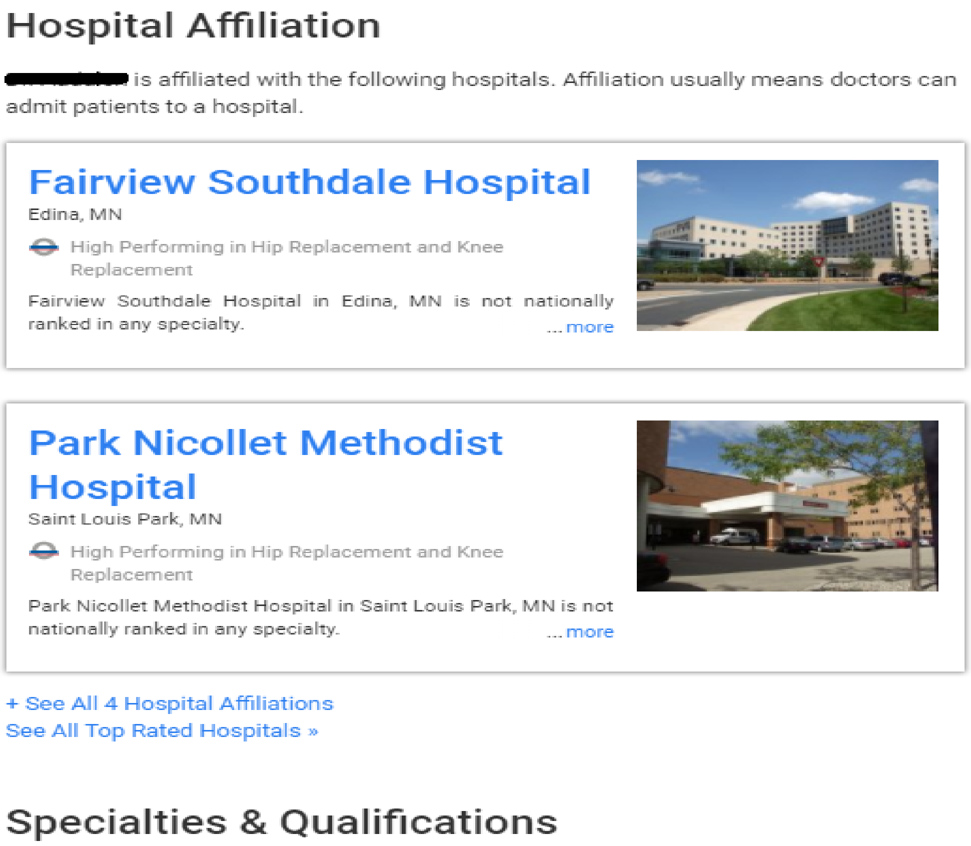

Physician with two affiliations

Figure S1. Examples of Physicians' pages listed through U.S. News & World Report with differing numbers of hospital affiliations.

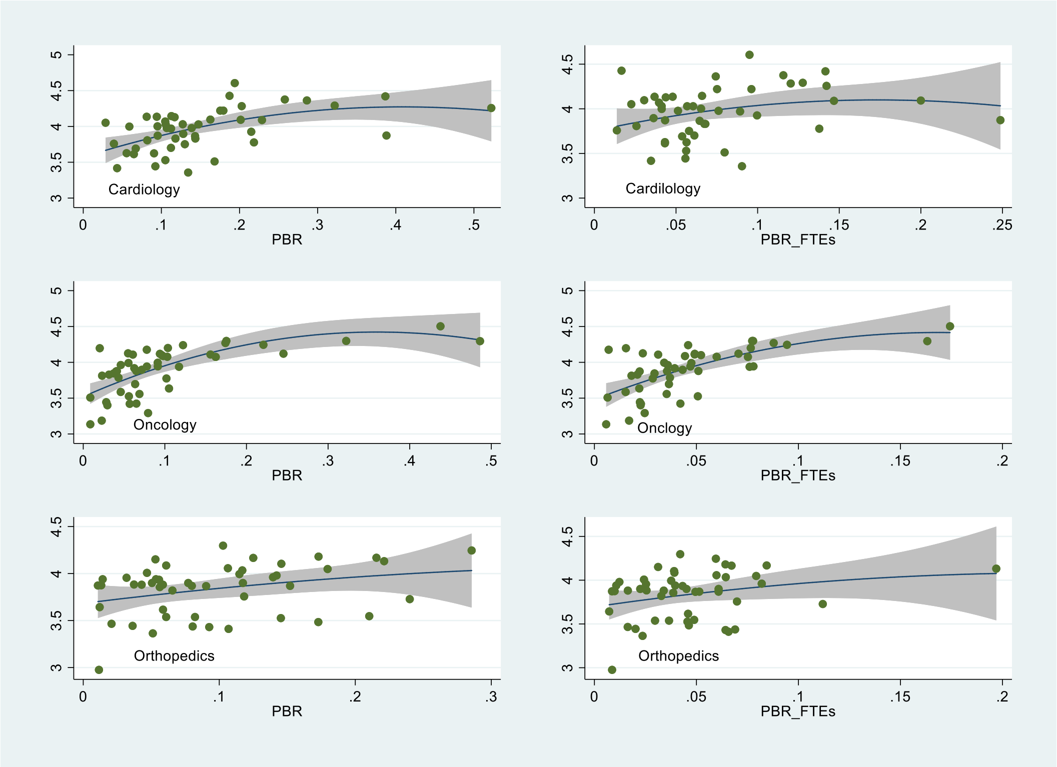

Figure S2. Predicted vs. observed values of ln(IHQ) as a function of PBR and PBR_FTEs without outliers. Caption: Predicted ln(IHQ) are in solid black lines. Observed ln(IHQ) are in green dots. Shaded areas represent 95% confidence intervals. All other variables are held at means.
